# Effect of neurogenic bowel dysfunction symptoms on quality of life after a spinal cord injury

**DOI:** 10.1186/s13018-023-03946-8

**Published:** 2023-06-26

**Authors:** Fater A. Khadour, Younes A. Khadour, Jiang Xu, Ling Meng, Lixin Cui, Tao Xu

**Affiliations:** 1 Department of Rehabilitation, Tongji Hospital, Tongji Medical College, Huazhong University of Science and Technology, 1095#, Jie-Fang Avenue, Qiaokou District, Wuhan, 430030 Hubei China; 2 Department of Rehabilitation, Faculty of Medicine, Al Baath University, Homs, Syria; 3 Department of Physical Therapy, Al Baath University, Homs, Syria; 4 Department of Physical Therapy, Physical Therapy Department for Neuromuscular and Neurosurgical Disorder and Its Surgery, Cairo University, Cairo, Egypt

**Keywords:** Spinal cord injury, Bowel dysfunction, Quality of life, Bowel management

## Abstract

**Background:**

Neurogenic bowel dysfunction (NBD) is a common problem among people with spinal injury; management of bowel dysfunction and related problems are considered significant factors in daily life after injury. But despite the critical relevance of bowel dysfunction in the daily life of SCI survivors, there have been few published studies on the management of NBD. So, this study aimed to describe the bowel programmers utilized by people with SCI in China and the impact of bowel dysfunction on the quality of life (QoL).

**Design:**

A cross-sectional online survey.

**Setting:**

Rehabilitation Medicine Department of Wuhan’s Tongji Hospital.

**Participants:**

SCI patients who had been diagnosed with neurogenic bowel dysfunction and who were receiving regular medical monitoring at the rehabilitation medicine department were invited to participate in our study.

**Outcome measures:**

A neurogenic bowel dysfunction (NBD) score is a questionnaire developed to evaluate the severity of neurogenic bowel dysfunction. A Short Form-12 (SF-12) was designed to measure the quality of life in people with SCI. Demographic and medical status information was extracted from their medical records.

**Results:**

The two questionnaires were sent to 413 SCI patients. Two hundred ninety-four subjects (43.1 ± 14.5 years of age; men, 71.8%) responded. Most of the respondents performed their bowel movement daily 153 (52.0%), a defecation time was 31–60 min among 70 (23.8%) of them, 149 (50.7%) used medication (drops or liquid) to treat constipation, and 169 (57.5%) used digital stimulation more than once per week to boost the bowel evacuation. This study found a significant association between the QoL score and the time used for each defecation, autonomic dysreflexia (AD) symptoms, taking medication to treat fecal incontinence, using digital stimulation, having uncontrollable flatus and perianal skin problems.

**Conclusion:**

Management of bowel dysfunction is complex and associated with QoL in people with SCI. Items of the NBD questionnaire that greatly deteriorated the QoL were time in one defecation > 60 min, symptoms of AD during or before defecation, taking medication (drops or liquid), and using digital stimulation. Dealing with those problems can improve the life quality of spinal cord injury survivors.

## Introduction

After a spinal cord injury (SCI), people may experience not only sensory and motor deficits, but also autonomic dysfunction (AD) which compromises respiratory, cardiovascular, urinary, gastrointestinal, and sexual functioning. Among the most common complications associated with SCI is neurogenic bowel dysfunction (NBD) [1]. NBD can include some or all changing intestinal motility, losing control of internal and external sphincters, and losing the ability to increase intra-abdominal pressure [2]. As a result, people with NBD may experience a range of symptoms, such as fecal incontinence, constipation, abdominal pain, and long-term complications, such as prolapses, anal fissures, hemorrhoids, and perianal skin problems [3]. The pathophysiology of NBD after an SCI is quite well studied [4]. It has been proposed that the defecation disorders often seen after complete or incomplete SCI could be due to a loss of sensory or motor control of the anorectum or/and pelvic floor, resulting in fecal incontinence or constipation, or both [5]. Certainly, SCI often leads to substantially prolonged colon transit times (CTTs) in both its chronic and acute phases [6]. A follow-up study found that supraconal SCI led to general colonic dysfunction within the first year after injury while chronic conal/cauda equina lesions led to considerable dysfunction in the rectosigmoid section [7]. Most patients experience delayed CTTs, which leads to constipation [8]. In contrast, fecal incontinence, if it occurs, arises from a loss of visceral perception and/or an inability to actively contract the external anal sphincter [9].

Managing bowel dysfunction and its related problems is usually an important factor in daily life after a spinal cord injury, affecting reintegration into society [10], and finding a suitable job [11]. Techniques to facilitate defecation include medication such as oral laxatives, prokinetics, or secretagogues, suppositories, helpful maneuvers such as manual evacuation, digital stimulation, abdominal massage or anorectal stimulation, or even additional surgical treatment when conservative treatments fail [2]. But despite the critical relevance of bowel dysfunction in the daily life of SCI survivors, there have been few published studies on the topic. Little research has focused on the management of NBD and evaluated the impact of bowel dysfunction on the QoL of Chinese patients with SCI [12]. Therefore, the purpose of this study was to describe the bowel programs utilized by patients with SCI in China and the impact of bowel dysfunction on the QoL of those patients.

## Methods

### Participants

The study included SCI patients who had received treatment at Tongji Hospital, a teaching hospital associated with Huazhong University of Science & Technology in Wuhan, China. The dataset included all SCI patients 18 or older with an NBD treated between February 2016 and January 2021. All had been diagnosed with a neurogenic bowel dysfunction lasting at least 6 months. There were 413 such former patients. All were sent the questionnaire, but only 294 agreed to participate in the study.

### Data collection

An online questionnaire was used to collect quantitative data using a cross-sectional design. Before receiving the questionnaire, each participant received a brief explanation of the study and its intent. Two nurses contacted the patients to encourage compliance. The process of data collection took about 2 months (from mid-September through November of 2021). The ethics committee of Tongji Hospital approved the study’s protocols.

### Instrument

All patients completed the following two questionnaires Neurogenic Bowel Dysfunction score (NBD Score) and Short Form-12 (SF-12) questionnaire.

The NBD score is a questionnaire developed for patients with SCI. It consists of 10 items that cover the following problems, frequency of bowel movements, headache, perspiration, or discomfort before or during defecation, tablets, and drops against constipation, time used for each defecation, frequency of digital stimulation or evacuation, frequency of fecal incontinence, medication against fecal incontinence, flatus incontinence, and perianal skin problems [13].

The Short Form-12 is an abbreviated version of the original 36-item QOL questionnaire, available in Chinese [14]. It comprises 12 items with a mental component summary (MCS) and a physical component summary (PCS). Possible scores for mental and physical health-related QOL subscales range from 0 to 100 points, and a higher score is interpreted as better QOL.

### Statistical analysis

Data analyses were carried out using version 25.0 of the SPSS software suite. The variables were investigated using a Kolmogorov–Smirnov test. In reporting descriptive statistics, normally distributed continuous data were expressed as mean ± standard deviation (SD), while the non-normally distributed continuous data were presented as the median (25th, 75th percentile). Categorical and nominal variables were presented as frequencies and percentages (%). Univariable analysis was computed to analyze the significance of any associations between the Items of Neurogenic Bowel Dysfunction questionnaire and SF-12 score. Variables with significant univariable effects were further processed using logistic multivariate regression analysis. Odds ratio (OR) is presented with a 95% confidence interval (CI). Microsoft Excel (Microsoft Office Package 13) was used for building tables. The level of significance was fixed at 0.05.

## Results

### Demographic and injury information

The response rate was 71.2% (*n* = 294), with a mean age of 43.1 ± 14.53 years. Furthermore, the 30–39 years age group had the largest 133 (45.2%), followed by 40–49 years 82 (27.9%). Most of the respondents were male 211 (71.8%), and 83 (28.2%) were female. Thus, the male-to-female ratio was 3:1. Concerning educational status, 144 (49.0%) of the respondents finished secondary school. Of the participants, 161 (54.8%) were from rural areas. Regarding occupational status, the most frequent occupational was a worker 116 (39.5%), followed by farmer 63 (21.4%).

In terms of injury characteristics, the most frequent level of injury was thoracic 122 (41.5%), followed by cervical 83 (29.3%). More than half of the respondents had an incomplete injury 173 (58.8%) and 121 (41.2%) a complete injury, in addition most of the injuries among the respondents resulted from traumatic reasons 253 (86.1%), the detailed demography is shown in (Table 1).

**Table 1:** Demographic and injury information (*n* = 294)

Category	*N* (%)
*Age (Years)*	
18–29 years	64 (21.7%)
30–39 years	133 (45.2%)
40–49 years	82 (27.9%)
50–59 years	9 (3.1%)
≥ 60	6 (2.1%)
*Gender*	
Male	211 (71.8%)
Female	83 (28.2%)
*Educational level*	
Primary	75 (25.5%)
Secondary	144 (49.0%)
Bachelor	53 (18.0%)
Higher	22 (7.5%)
*Occupation*	
Farmer	63 (21.4%)
Worker	116 (39.5%)
Government offices	31 (10.6%)
Retired	16 (5.4%)
Students	19 (6.4%)
Others*	49 (16.7%)
*Injury level*	
Cervical	83 (29.3%)
Thoracic	122 (41.5%)
Lumbar	73 24.8%)
Sacral	13 (4.4%)
*Completeness of injury (%)*	
Complete	121 (41.2%)
Incomplete	173 (58.8%)
*Causes of injury*	
Truman	253 (86.1%)
Disease involving the spinal cord	41 (13.9%)
*Place of residence*	
Urban	133 (45.2%)
Rural	161 (54.8%)

*SCI* spinal cord injury, and other* included unemployed individuals and self-employed individuals

### Outcomes of bowel management

52.0% of the respondents (*n* = 153) carried out at least once per day. Seventy respondents (23.8%) said it took 31–60 min, with 46 (15.6%) reporting taking longer. Additionally, 56.8% of the respondents (*n* = 167) reported that they had experienced symptoms of autonomic dysreflexia (such as, headache, perspiration, or discomfort) before or during defecation. Most respondents reported treating their constipation using drops or liquid, while only 51 (17.3%) took medication (tablets) to manage their constipation.

Regarding evacuation techniques, half of the respondents used digital stimulation once or more per week to evacuate their bowels 169 (57.5%). In contrast, about a third of the sample, 95 (32.3%), experienced involuntary defecation 3–4 times a month. Concerning fecal incontinence, only 52 (17.7%) respondents reported using medication to treat fecal incontinence. About uncontrollable flatus, it is found only in 81 (27.6%) respondents. About a third of the sample experienced 93 (31.6%) perianal skin problems caused by performing digital stimulation and different types of medication to help bowel evacuation.

Table 2 shows that there was no significant association between the Qol results and frequency of bowel movements of the respondents. There were, however, significant relationships between the Qol results and the time consumed during defecation (*p* < 0.001) and symptoms of autonomic dysreflexia (*p* = 0.021). Additionally, there was no significant association between the Qol results and fecal incontinence, but the association with taking medication (drops or liquid) (*p* = 0.018) or use of digital stimulation to treat constipation (*p* < 0.001), as well as existence a significant association with uncontrollable flatus and perianal skin problems (*p* = 0.03,* p* = 0.043, respectively).

**Table 2:** Demonstrates the impact of NBD on daily life (*n* = 294)

Items of the NBD questionnaire	*N* (%)	QoL score	
		*p*-Value	OR (95% CI)
*Frequency of bowel movements*			
Daily	153 (52.0%)	0.082	
2–6 times per week	106 (36.1%)		
Less than once per week	35 (11.9)		
*Time used for each defecation*			
Less than 30 min	**178 (60.6%)**	** < 0.001**	1
31–60 min	**70 (23.8%)**		1.8 (1.3–3.4)
More than an hour	**46 (15.6%)**		2.3 (1.4–3.9)
*Symptoms of headache, perspiration, or discomfort before or during defecation*			
No	**127 (43.2%)**	**0.021**	1
Yes	**167 (56.8%)**		1.4 (1.2–3.5)
*Taking medication (tablets) to treat constipation*			
No	243 (82.7%)	0.091	
Yes	51 (17.3%)		
*Taking medication (drops or liquid) to treat constipation*			
No	**145 (49.3%)**	0.018	1
Yes	**149 (50.7%)**		1.8 (1.6–2.7)
*Frequency of digital stimulation*			
Less than once per week	**112 (42.5%)**	** < 0.001**	1
Once or more per week	**169 (57.5%)**		1.7 (0.9–2.6)
*Frequency of involuntary defecation*			
Daily	43 (14.6%)	0.103	
1–6 times a week	111 (37.8%)		
3–4 times a month	95 (32.3%)		
A few times a year or less	45 (15.3%)		
*Taking medication to treat fecal incontinence*			
No	242 (82.3%)	0.24	
Yes	52 (17.7%)		
*Having experienced uncontrollable flatus*			
No	**213 (72.4%)**	**0.03**	
Yes	**81 (27.6%)**		
*Having perianal skin problems*			
No	**201 (68.4%)**	**0.043**	
Yes	**93 (31.6%)**		

Bold numbers indicate significant results at *p* < 0.05

*OR* odds ratio, *CI* confidence interval

Six factors with significant differences in the univariate analysis were further together analyzed by logistic multivariate regression analysis to acquire adjusted OR, including the time consumed during defecation, symptoms of autonomic dysreflexia (AD), taking medication (drops or liquid) or use of digital stimulation to treat constipation, uncontrollable flatus, and perianal skin problems. The results revealed that the QoL was more affected by the following four factors, time consumed during defecation, symptoms of autonomic dysreflexia, taking medication (drops or liquid), or using digital stimulation to treat constipation. The more consuming time while managing the bowel, the more decrease in the QoL (OR 2.3, 95% CI 1.4–3.9), and the impact of NBD was more common among patients who had symptoms of autonomic dysreflexia (OR 1.4, 95% CI 1. 2–3.5). Lowering in the QoL results was more common among respondents taking medication (drops or liquid) or using digital stimulation more than once per week to treat constipation (OR 1.8, 95% CI 1.6–2.7), (OR 1.7, 95% CI 0.9–2.6), respectively (Table 2).

## Discussion

Neurogenic bowel dysfunction (NBD) and associated complications among people with SCI were increasingly recognized as significant factors in the quality of life and community reintegration [15–18]. Furthermore, bowel dysfunction significantly impacts the QoL more than bladder function, wheelchair use, and pain [16]. Although the importance of this problem, there are only a few studies about NBD among Chinese people with SCI [19]. So, this study aimed to highlight the management of NBD and its impacts on people with SCI who received treatment at Tongji Hospital from 2016 to 2021.

The current findings showed that about half of the respondents conducted their bowel management once per day and these are consistent with a study conducted by Inskip et al. [20] where 41% of the sample carried out bowel management daily, in contrast, other study conducted by Kim [21] showed that only 21% of the sample managed their bowel movement daily. In clinical practice, the importance of frequent and regular bowel care should be emphasized to avoid problems such as constipation, abdominal pain, and abdominal distension. Constipation is often associated with a lack of routine and infrequent management [5]. This study revealed also that about 24% of the respondents spent between 31 and 60 min daily on their bowel routine, and that almost 15% spent more than 60 min agrees closely with the findings of another study in the Republic of Korea where 26% of the patients spent 31–60 min, and 10% spent more than 60 min [21]. Time consumed during bowel care is crucial because it is closely related to life satisfaction and QoL [20] with the study finding a significant association between the time used for each defecation and the QoL score. The more time needed during bowel management, the lower the QoL scores; this result consistent with previous studies [20, 21].

Bowel dysfunction is the second most common cause of autonomic dysreflexia [22], and bowel management has been identified as a stimulus for AD among people with SCI [23]. Symptoms of autonomic dysreflexia (such as headache, perspiration, or discomfort) from the gastrointestinal tract accounted for about 44% of chronic SCI patients, affecting QoL and lifestyle [24]. Symptoms of possible autonomic dysreflexia were found in about half of the respondents in this study, and there was a significant association with QoL. In contrast, a study conducted by Kim et al. showed that there was no association between the symptoms of AD and a decrease in Qol score; this can be explained by the fact that the study conducted by Kim et al. [21] was used self-reported questionnaire, examples given as headache, sweating, or discomfort might be read as minor symptoms rather than those AD.

For people living with a spinal cord injury, the effective management of bowel dysfunction and its related problems is crucial, as it can significantly impact their quality of life. Our results presented that the bowel management techniques described were multifactorial and complicated. About half of the respondents reported taking medication (drops or liquids) to treat their constipation, and half of the sample also reported using digital stimulation more than once per week. These results agree with the findings of similar previous studies [21, 25, 26]. In addition, our results showed a significant association between the methods used to boost bowel evacuation and Qol, where the more used medication (drops or liquids) and digital stimulation, the lower QoL of the respondents. The striking thing in this study was that 17.7% of the respondents took medication to treat fecal incontinence, and this rate is considered high compared with previous studies [12, 13, 16, 27]. This high rate of fecal incontinence may be because of the unwise use of unsuitable techniques [28]. Fecal incontinence can be appeared after using medication (drops, liquids, or tablets) to deal with constipation [20]. About 28% of the respondents had flatus incontinence and was associated with QoL. These results are consistent with previous studies, which showed that flatus incontinence was associated with lower rates of QoL [17, 29].

As well as the results of this study revealed that perianal skin problems were common among the respondents; one-third of the sample said they experienced them, and the perianal skin problems were associated with Qol. These results are in line with a study conducted by Kim et al. [29]; in contrast, the other studies conducted in Canada [20] and Malaysia [27] did not show any association between perianal skin problems and Qol, this may be attributed to the severity of SCI patients who participated in those studies, where these problems often occur after high-level SCI [4]. At the same time, the perianal skin problems that result from bowel care (such as, hemorrhoids, anal fissure, and rectal prolapse) are most common among people with complete and incomplete cervical injuries [30]. Those patients have SCI lesions above the conus and retain age anocutaneous reflexes, so we can use this reflex to stimulate bowel evacuation [31]. A pharmacological approach administered via rectal and using some techniques that increase bowel evacuation, such as digital rectal evacuation, is most common among people with SCI who have upper motor neuron bowel dysfunction (UMNBD) [32]. So, perianal skin problems are very common among those patients because of the usage frequency of these medications and the technical methods to boost bowel evacuation [30].

Before closing, it is important to call attention to this study’s major limitations. It was based in one hospital in one city in China. All of the participants had been treated in the same rehabilitation department. So, the sample may not represent the entire cohort of community-dwelling spinal cord injury survivors in China. Among them, only former patients with an email address on file were included. That tended to exclude China’s vast population of persons with limited literacy. As a questionnaire-based study, it depended on the respondents' memory of their bowel care, possibly injecting some recall bias. Future studies should focus on a qualitative study design to give us a deeper understanding and better information about the problems in this population concerning neurogenic bowel care.

## Conclusion

Managing neurogenic bowel dysfunction requires numerous interventions, demands substantial time, and considerably impacts the quality of life of community-dwelling SCI survivors in China, as previously reported in other countries. Bowel management-related factors influencing the QoL included defecation time > 1 h, AD symptoms before or during defecation, and taking medication (drops or liquid) or digital stimulation to treat constipation. One way to overcome this challenge may be to reduce perianal skin problems, taking medication (drops or liquid) or digital stimulation, and symptoms of AD. Efforts to reduce the time taken in defecation may also be needed to improve QoL among those people. In addition, to increase societal awareness of this problem and further management for people with SCI may be required.

## Data Availability

The original contributions presented in the study are included in the article/supplementary material, further inquiries can be directed to the corresponding author.
